# Immunomodulation of Macrophages in Diabetic Wound Individuals by Structurally Diverse Bioactive Phytochemicals

**DOI:** 10.3390/ph17101294

**Published:** 2024-09-28

**Authors:** Krishnendu Adhikary, Riya Sarkar, Sriparna Maity, Ishani Sadhukhan, Riya Sarkar, Krishnendu Ganguly, Saurav Barman, Rajkumar Maiti, Sanjoy Chakraborty, Tandra R. Chakraborty, Debasis Bagchi, Pradipta Banerjee

**Affiliations:** 1 Department of Interdisciplinary Science, Centurion University of Technology and Management, Khurda 752050, Odisha, India; krisskrishnendu@gmail.com; 2 Department of Medical Lab Technology, Dr. B. C. Roy Academy of Professional Courses, Bidhannagar, Durgapur 713212, West Bengal, India; 3 Department of Food Processing, Indian Institute of Engineering Science and Technology, Shibpur, Howrah 711103, West Bengal, India; 4 Department of Medical Lab Technology & Biotechnology, Paramedical College Durgapur, Durgapur 713212, West Bengal, India; 5 Department of Soil Science, Centurion University of Technology and Management, Paralakhemundi 761211, Odisha, India; 6 Department of Physiology, Bankura Christian College, Bankura 722101, West Bengal, India; rajkumar@bankurachristiancollege.in; 7 Department of Biological Sciences, New York City College of Technology, City University of New York (CUNY), Brooklyn, NY 11201, USA; 8 Department of Biology, College of Arts and Sciences, Adelphi University, Garden City, NY 11530, USA; 9 Department of Psychology, Gordon F. Derner School of Psychology, Adelphi University, Garden City, NY 11530, USA; 10 Department of Pharmaceutical Sciences, College of Pharmacy and Health Sciences, Texas Southern University, Houston, TX 77004, USA; 11 Department of Surgery, University of Pittsburgh, Pittsburgh, PA 15213, USA

**Keywords:** diabetic ulcer, phytochemical, miRNA, chronic wound, tissue repair

## Abstract

Diabetes-related ulcers and slow-healing wounds pose a significant health risk to individuals due to their uncertain causes. Mortality rates for diabetes foot ulcers (DFUs) range from 10% after 16 months to 24% after five years. The use of bioactive phytochemicals can play a key role in healing wounds in a predictable time. Recent literature has demonstrated that various natural substances, including flavonoids, saponins, phenolic compounds, and polysaccharides, play key roles at different stages of the wound-healing process through diverse mechanisms. These studies have categorized the compounds according to their characteristics, bioactivities, and modes of action. In this study, we evaluated the role of natural compounds derived from plant sources that have been shown to play a crucial role in immunomodulation. Macrophages are closely involved in immunomodulation within the wound microenvironment and are key players in efferocytosis, inflammation resolution, and tissue regeneration, all of which contribute to successful wound healing. Phytochemicals and their derivatives have shown capabilities in immune regulation, including macrophage migration, nitric oxide synthase inhibition, lymphocyte and T-cell stimulation, cytokine activation, natural killer cell enhancement, and the regulation of NF-κβ, TNF-α, and apoptosis. In this review, we have studied the role of phytochemicals in immunomodulation for the resolution of diabetic wound inflammation.

## 1. Introduction

**Figure 1:**
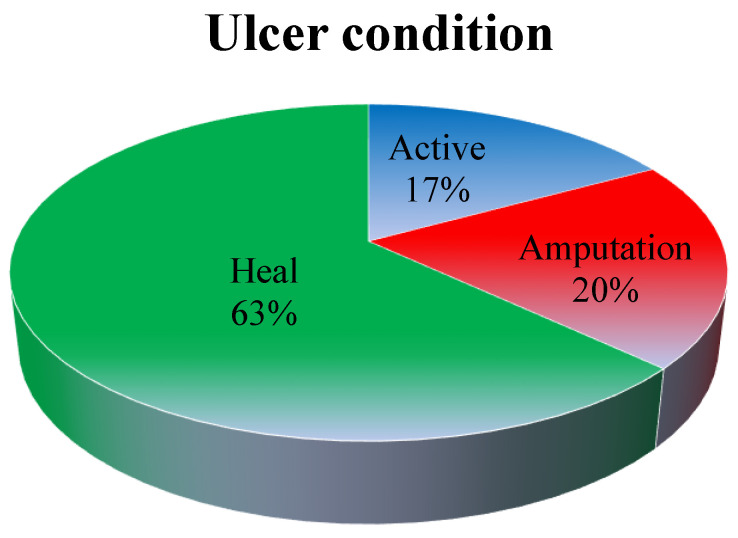
Different phases of ulcer conditions, including active to healing conditions: healed, 63%; amputation, 20%, and active wounds, 17% (figure created by MS PowerPoint 2007).

The human body faces continuous exposure to diverse agents that stimulate the production of reactive oxygen species (ROS), termed free radicals. These radicals, through the transfer of their unpaired electrons, instigate oxidation within cellular machinery. An imbalance between ROS and antioxidants can lead to the onset of a condition termed “oxidative stress”, which contributes to the development of various pathological conditions, including diabetes [1]. Diabetes mellitus (DM) encompasses a range of metabolic and physiological irregularities characterized by elevated blood glucose levels. This elevation typically stems from either insufficient insulin secretion by pancreatic β-cells or decreased sensitivity of insulin receptors, impairing the proper utilization of blood glucose for energy [2]. DM complications can be broadly categorized into two main types: microvascular complications, which affect small blood vessels in the kidneys, peripheral nerves, and retina, leading to diseases such as nephropathy, neuropathy, and retinopathy, respectively; and macrovascular complications, which impact larger blood vessels including those in cerebrovascular disease, coronary artery disease, and peripheral artery disease [3]. Diabetic ulcers are also a potential issue linked to diabetes, marked by stubborn open sores or wounds on different areas of the skin that have difficulty healing. Diabetes patients with foot and leg ulcers exhibited a lower 5-year survival rate (43% vs. 68%) compared to non-diabetic patients with ulcers (56% vs. 68%) and the general population as a whole. After a median follow-up period of 16 months, the reported fatality rate for persons with diabetic foot ulcers (DFUs) ranges from around 10% to 24% after five years. These ulcers usually arise from minor injuries that, because of factors such as nerve damage and impaired healing mechanisms, may be overlooked and progress into a more severe state. Wounds are classified into two forms based on their healing potential: chronic and acute [4]. Chronic wounds encompass tissue injuries that do not follow a structured sequence of healing stages and require more than ten to twelve weeks to heal (Figure 1). Bacteria play a pivotal role in initiating wound infections among diabetic patients [5]. In severe cases, limb amputation may be necessary due to diabetic complications. Consequently, broad-spectrum antibiotics are often employed for antibacterial therapy. However, indiscriminate antibiotic usage over time can contribute to developing resistant strains against multiple antibiotics. These antibiotic agents, alongside their constrained tolerability, adverse effects, and expenses, have hastened the pursuit of alternative medications offering enhanced efficacy, potency, and diminished side effects [6]. Notably, numerous contemporary therapeutic agents in conventional medicine originated from medicinal plants.

Moreover, factors like bacterial resistance, environmental decline, and pollution, alongside the indiscriminate use of conventional medicines, have spurred a resurgence in exploring medicinal plants as effective and safer alternatives for managing diverse infections like diabetic wounds. Medicinal plants are renowned for their abundance of alkaloids and other phytochemical compounds, which can be potent antibacterial agents for treating various ailments [7]. The discovery of bioactive compounds in plants has led scientists to explore medicinal plants’ potential wound-healing properties and isolate chemicals linked to wound healing. A few studies are available on possible bioactive compounds and their therapeutic potential on chronic wound healing and molecular mechanisms of repair processes. The current study explores the role of phytochemicals in the modulation of resident macrophages in diabetic ulcer/wound repair. This review also identifies new avenues for future research in phytochemical studies in diabetes-induced wound repair and tissue regeneration, especially in shortening the wound closure duration and macrophage modulation.

## 2. Method

### 2.1. Search Strategy

A systematic literature review from 1999 to 2024 was conducted using the Google Scholar, Web of Science, Scopus, and PubMed databases, along with the Preferred Reporting Items for Systematic Reviews and Meta-Analysis. The search included appropriate English keywords such as ‘diabetic ulcer’, ‘phytochemical’, ‘miRNA’, ‘nanoparticle’, ‘wound repair’, ‘activated macrophage’, ‘antioxidants’, ‘angiogenesis’, ‘apoptosis’, ‘efferocytosis’, ‘inflammation’, and ‘vasodilation’. There were no constraints.

### 2.2. Study Selection Criteria

The articles included in the study focused on various aspects related to phytochemical or bioactive compounds, such as their sources and types, metabolic pathways, and their role in treating and managing diabetic wounds. Additionally, the articles also explored the pathogenesis and immunology of diabetic ulcers, the involvement of macrophages in wound resolution, and their molecular activation and plasticity, as well as the use of nanoparticles for delivering therapeutic drugs for diabetic ulcer healing. The exclusion criteria included synthetic particles, lab-synthesized artificial chemicals, and chemical materials different from natural or herbal ones. Both replicated studies and papers authored by the same individual were excluded if they were identical. Articles and other works that did not provide new or updated processes were also ignored. The authors employed inclusion and exclusion criteria to assess the titles and abstracts individually. We obtained complete copies of relevant research. Upon reviewing the references of the retrieved papers, more articles were selected. Conflicts among reviewers were resolved by deliberation and agreement.

### 2.3. Data Extraction

In vivo and translational wound models, macrophage-specific deletions, techniques for differentiating macrophage subsets, and the properties, bioactivities, and modes of action of natural bioactive compounds and phytochemicals have all been identified in the data gathered for these studies. Data on the dosage, duration of wound contraction, mechanism of action, and manner of administration of phytochemicals for each mouse model were added to the original publication.

### 2.4. Results

The first search results showed a total of 1012 items. An additional 38 publications were found when a search was conducted for relevant citations in the reference section of the first study. Accordingly, a total of 1050 records were found. Following a rigorous review of the titles and abstracts, 434 articles were selected for further investigation. After completing the comprehensive content analysis, 222 of these items were deleted with reason (the criteria for removal are specified in the Methods section). The final comprehensive synthesis, which totals 212 study results, is presented here.

## 3. Source and Types of Phytochemical or Bioactive Compounds

**Figure 2:**
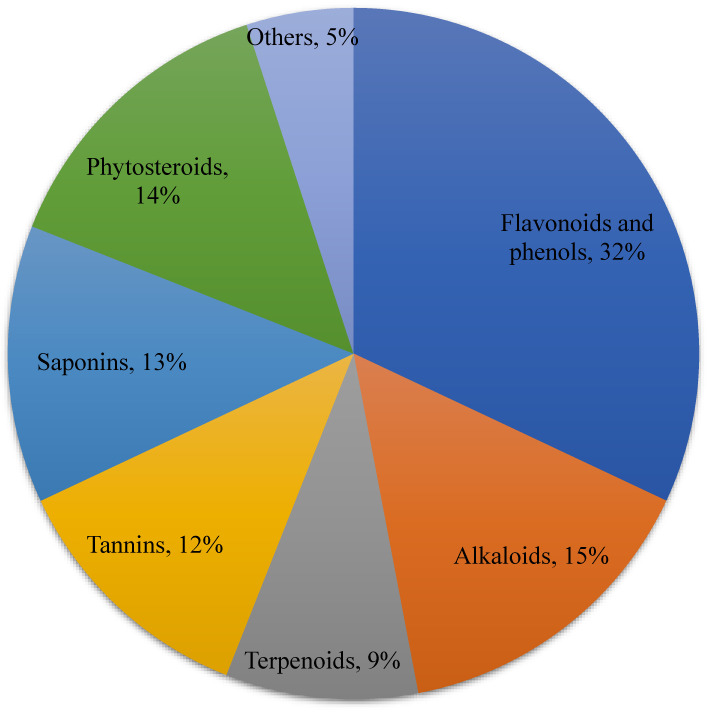
Different bioactive compounds with antidiabetic properties are available in plants. Different bioactive compounds are present in plants with the following percentages: flavonoids and phenols, 32%; alkaloids, 15%; terpenoids, 9%; tannins, 12%; saponins, 13%; phytosteroids, 14%, and others, 5% (figure created by MS PowerPoint 2007).

Ethnobotanical surveys have revealed many plant species with wound-healing properties across Africa and many developing regions. The application of herbal plants in ulcer/wound healing includes disinfection, debridement, and establishing an ideal environment to support the natural healing process (Figure 2).

**Figure 3:**
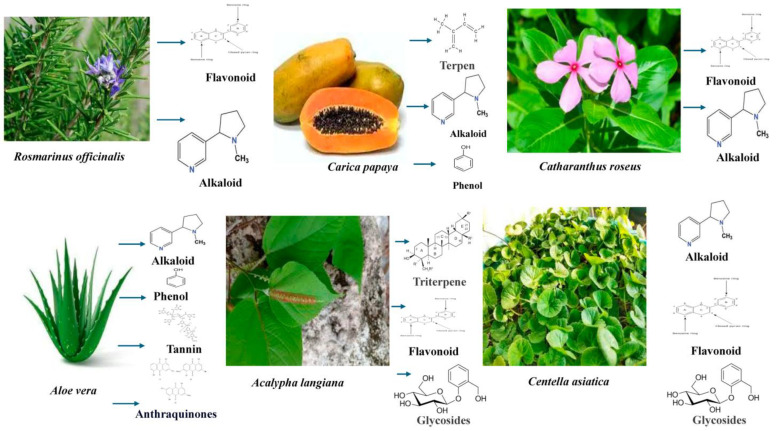
Some medicinal plants and their active phytochemicals help to heal diabetic wounds (Figure created by MS PowerPoint 2007).

The delayed cure of ulcers/wounds related to diabetes is a major health concern for healthcare professionals globally, particularly because of their unclear causes in some instances. Therefore, a therapeutic approach to tackle this issue involves the use of medicinal plants, especially in regions with limited resources (Figure 3 and Table 1) [8,9,10].

## 4. Phytochemicals Regulate Human Immune System and Metabolic Pathways

The treatment of diabetic ulcers and wounds with phytochemicals involves complex metabolic pathways that contribute to wound healing through various mechanisms, including anti-inflammatory, antioxidant, antimicrobial, and angiogenic effects. A polyphenol derived from the Indian dietary spice curcumin inhibits the nuclear factor-kappa B (NF-κβ) pathway, reducing the production of pro-inflammatory cytokines like tumor necrotic factor-α (TNF-α), interleukin-1β (IL-1β), and interleukin-6 (IL-6). Activator Protein 1 (AP-1), mitogen-activated protein kinases (MAPKs) [25,26], nuclear factor kappa-B (NF-κB), and other signaling pathways are all modulated by curcumin via its interactions with Toll-like receptors (TLRs). In addition to offering a possible cure for inflammatory diseases, this aids in regulating the generation of inflammatory mediators. Curcumin interacts with PPARγ, a receptor activated by the peroxisome proliferator, to reduce NF-κB activity [27]. Curcumin can reduce inflammation by influencing the inflammatory signaling pathway involving Janus kinase and signal transducer and activator of transcription (JAK/STAT) [28,29]. Additionally, the cytoplasmic complex known as the NOD-like receptor pyrin domain-containing 3 (NLRP3) inflammasome, made up of several proteins, is essential to developing numerous inflammatory illnesses. The protease caspase-1, an apoptosis-associated speck-like protein with a caspase recruitment domain, and a sensor protein make up the NLRP3 complex. Curcumin may either directly decrease the NLRP3 inflammasome’s development or stop it from activating by blocking the NF-κB pathway. This may be one of the ways curcumin relieves inflammatory conditions [30,31].

Curcumin, a bioactive molecule produced by the turmeric plant, has demonstrated substantial antioxidant effects, making it a viable agent for treating diabetic foot ulcers (DFUs) [32]. The pathophysiology of DFUs frequently involves persistent inflammation and poor wound healing, mostly owing to oxidative stress induced by the overproduction of reactive oxygen species (ROS) [33]. ROS, which include hydrogen peroxide (H_2_O_2_) and superoxide (O_2_^•−^are natural by-products of aerobic respiration that play critical roles in cell development, death, intracellular signaling, differentiation, and immunology [34]. However, high ROS levels can cause oxidative damage such as DNA breakage, enzyme inactivation, and lipid peroxidation, all impeding proper wound healing [35]. During wound healing, ROS play two roles. On the one hand, they serve as the immune system’s protection mechanism against microbes. Moreover, their extended presence and high concentrations cause oxidative stress, which damages human cells and prevents tissue rebuilding. The inflammation seen during DFU wound healing is mostly caused by oxidative stress. Consequently, ROS must be adequately controlled to prevent protein damage in tissues. Catalase, glutathione peroxidase, and superoxide dismutase are antioxidant enzymes that protect human cells from damaging ROS. Topical antioxidants with free radical-scavenging properties can considerably enhance wound healing [36]. Picroliv, a standardized fraction derived from the plant *Picrorhiza kurroa*, has significant active components such as iridoid glycosides, picroside-1, and kutkoside. It has been used traditionally to treat various diseases, including fever, asthma, allergies, hepatitis, and inflammation [37].

Recent investigations have shown that microglia improve angiogenesis and wound healing in ex vivo rat aorta ring models. Picroliv was shown to increase endothelial cell sprouting and migration while also promoting re-epithelialization, neovascularization, and the migration of numerous cells into the wound bed, including endothelial cells, dermal myofibroblasts, and fibroblasts [38]. Additionally, during hypoxia, picroliv increased the production of vascular endothelial growth factor (VEGF) in human umbilical vein endothelial cells and insulin-like growth factor. Despite these findings, it is unknown what mechanism underpins picroliv’s therapeutic activities (Table 2) [39]. Arnebin-1, a chemical obtained from the roots of *Arnebia nobilis*, a *Boraginaceae* genus member, is also recognized for wound healing and has long been utilized in traditional Indian medicine [40].

## 5. Pathogenesis and Immunology of Diabetic Ulcer

**Figure 4:**
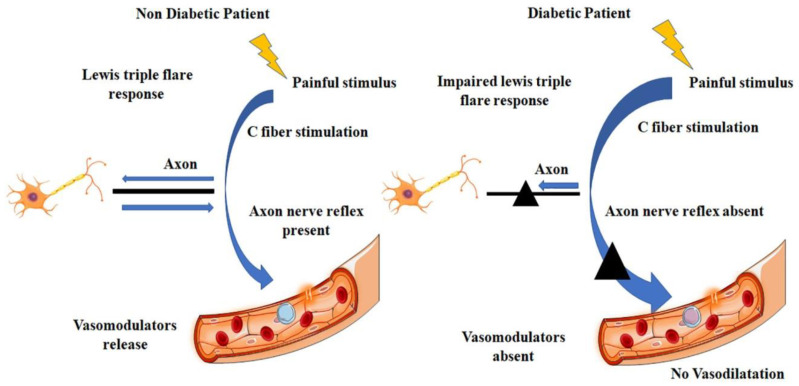
A comparison of diabetic people with non-diabetic participants with respect to injury and inflammation. In the case of non-diabetic patients, a painful stimulus governs the axon nerve reflex via C-fiber stimulation, leading to vasomodulator release to promoter vasodilation. In contrast, in diabetic patients, the axon nerve reflex is blocked and therefore, vasomodulators will not be released, leading to no vasodilation (figure created by BioRender).

DM is an intricate metabolic condition impacting over 340 million people globally. Among these individuals, approximately 20–25% encounter diabetic wounds, often concentrated on the foot, at some point in their lives [41]. These wounds, known as diabetic foot ulcers (DFUs), manifest as a prevalent complication of diabetes mellitus, stemming from a trio of factors: ischemia, peripheral neuropathy, and arteriopathy (Figure 4). Diabetes care, healthcare accessibility, other medical disorders, and the demography under study are some of the variables that affect the morbidity and mortality rates linked to DFUs. The formation of DFUs is a serious risk factor for people with diabetes mellitus since the precise mechanisms underlying their emergence are still poorly understood. A deeper comprehension of the several elements that contribute to their etiology can open the door to the development of cutting-edge therapies meant to improve the course of healing. In order to do this, this review aims to offer a platform to decipher the complexities of DFU etiology and investigate novel treatment modalities and drugs for treating this illness [42]. The natural healing process of wounds encounters numerous challenges during DFUs. These include reduced growth factor activity, cellular proliferation that is not as strong, elevated inflammatory marker levels, insufficient development of new blood vessels, and an imbalance in the production and degradation of extracellular matrix (ECM). Important participants in each of these processes are matrix metalloproteinases (MMPs). The review shows that DFUs usually show an overabundance of MMPs, which cause tissue breakdown and impair wound healing. The ability to precisely control MMP levels in DFUs may hasten and improve wound healing [43].

Furthermore, scientists noted that MMP-9 is particularly high in DFUs, indicating that it could be a useful target for therapy. Controlling bacterial growth becomes essential for promoting wound healing, given the high frequency of infections linked to DFUs [44]. Advanced nanotechnology produces nano-silver, a nanoparticle with strong skin permeability and strong antibacterial characteristics. Because it can promote epidermal cell growth and aid in blood vessel regeneration, epidermal growth factor, or EGF, has attracted attention in the field of wound care. Using nano-silver dressings in conjunction with EGF was very successful in controlling wound infections, preventing the growth of bacteria, speeding up the formation of granulation tissue and epidermal growth, and facilitating the healing process in general [45]. This creative method provides insightful information on handling DFUs. It is possible for stem cells to induce differentiation and release cytokines, which facilitate the development of a capillary network in ischemia tissues. Patients may benefit therapeutically from this procedure, which can create collateral circulation, improve blood perfusion, and lessen the ischemic state of afflicted tissues [46]. The primary risk factors for the formation of diabetic foot ulcers include peripheral neuropathy (motor, sensory, and autonomic), anatomical abnormalities, peripheral vascular disease, compromised immunity, and inadequate metabolic control. Social factors also come into play, including behavioral, psychological, and emotional problems [47]. Three distinct dimensions should be considered when evaluating risk factors for diabetic individuals’ development of foot ulcers: (a) the study of pathophysiology, (b) changes in anatomy and structure, and (c) impacts of the environment.

### Pathophysiology and Immune System Alteration

Biomolecular alterations are brought on by hyperglycemia, which triggers the onset of neuropathy. In the last twenty years, a significant body of information has been gathered to indicate the possible pathogenetic significance of several pathways that contribute to wound development in diabetics. The principal mechanisms consist of nerve ischemia or hypoxia, persistent oxidative damage, the polyol pathway being overactive, advanced glycation end-product levels being elevated, gamma-linolenic acid deficiency, an increase in the B-isoform of protein kinase C, cytokine dysfunction and deviations in collagen molecules (elastin, proteoglycans, etc.), deficiency in growth hormones, impaired mitochondrial activity, and malfunction of the endothelium [48]. 

Increased protease secretion is an additional contributing factor. Wound repair is generally a very well-coordinated procedure that includes the interplay of several previously mentioned components. Every stage of the recovery process depends on this coordinated endeavor. When tissue is injured, the injured area releases collagen and other stimuli quickly, which sets off a series of chemical, mechanical, biological, and physical reactions. These alterations can have molecular repercussions that result in peripheral vascular disease and even damage to nerve fibers. Endothelial dysfunction is a primary impediment to microcirculation and is characterized by altered vascular cell division, expansion of the basement membrane, reduced nitric oxide generation, increased blood viscosity, altered microvascular tone, and reduced blood flow [49]. Nonetheless, the immune system was weakened by reduced leukocyte activity, inappropriate inflammatory responses, and disruption of cellular immunity, which included fibroblast proliferation suppression and keratinocyte basal layer impairment. As a result, epidermal cell migration was reduced [50,51]. Furthermore, the impairment of the nerve axon reflex is a critical component influencing the microcirculation of the neuropathic foot. Adjacent fibers undergo retrograde stimulation in response to stimulation of C-nociceptive fibers [52]. Substance P (SP), calcitonin gene-related peptide (CGRP), neuropeptide Y (NPY), and histamine are among the vasodilators that these fibers quickly release, causing vasodilation (also known as the Lewis Triple Flair Response) [53].

The Lewis response mechanism comprises a series of reactions: capillary dilation causes a red spot to appear, and arteriolar dilation mediated by the axon reflex causes a flare, which is the redness spreading to the surrounding area. Ultimately, fluid exudation from capillaries and venules causes a wheal to form. Figure 4 shows this approach for patients with and without diabetes. Three types of morphological and structural changes associated with diabetic neuropathy are identified: sensory, motor, and autonomic.

## 6. Macrophages in Wound Resolution: Molecular Activation Mechanisms

Macrophages play a crucial role in wound healing, controlling tissue repair, eliminating cell debris, and reducing inflammation. The intricacy of macrophage action inside the wound is becoming more widely understood, and when macrophages are stimulated incorrectly, as in the case of fibrosis or chronic non-healing wounds, there can be negative consequences. Macrophage-specific deletions, novel methods to differentiate between subsets of macrophages, and advancements in in vivo and translational wound models have shown a wide range of macrophage activation and effector activities. Here, we provide an overview of the key players—cytokines, apoptotic cells, nucleotides, and mechanical stimuli—in the activation and activity of wound-healing macrophages. We highlight current research that shows these parameters work together to promote the best possible wound healing. The consequences for wound healing are then discussed. We present new methodologies, such as cell tracking and single-cell RNA sequencing, that have shown impressive versatility and variety in macrophages derived from blood or living in tissues. Finally, we assess the effect of decreased macrophage activity on aberrant wound healing associated with diabetes, aging, and fibrosis [54,55,56,57].

Blood clotting, inflammation reduction, and the formation of new blood vessels and tissue are all stages of wound healing. The process of repairing tissue and preserving stability after an infection or physical trauma is referred to as wound healing. These stages are common to all wounds, even though cells and molecules differ according to the organ (skin, lung, liver, or brain) and kind of damage (burn or infection) [58,59,60,61,62,63]. To repair wounds, macrophages are required. After a disease has been cured, they eradicate pathogens, repair tissue, and lessen inflammation. Pathogens are consumed and eliminated by the inflammatory macrophage, dead cells are removed, and inflammation is reduced by the resolving macrophage, and remodeling of tissue macrophage subsets may exhibit a range of macrophage activation depending on cell growth and extrinsic stimuli. The “M2” macrophages, also known as differentially triggered and anti-inflammatory macrophages, that are stimulated by T helper type 2 (Th2) cytokines were examined [64,65,66,67,68,69,70,71,72,73]. These macrophages are essential for reducing inflammation and encouraging tissue regeneration [74,75,76,77,78]. We will start by discussing newly discovered mechanisms that both initiate and regulate the activity of these cells. We will next discuss how ontogeny and plasticity contribute to the development of these unique macrophage subgroups that are engaged in wound healing. Lastly, we will discuss how the disease might be made worse by these cells not working properly. For therapeutic reasons, it would be extremely helpful to have a more thorough knowledge of these many groups of macrophages engaged in wound healing, including their activation mechanism and the chemicals they release, and to develop strategies to modify them to promote wound healing. Death might also come from inadequate wound healing [79,80,81,82,83].

### 6.1. Stimulation of Wound-Healing Macrophages

**Figure 5:**
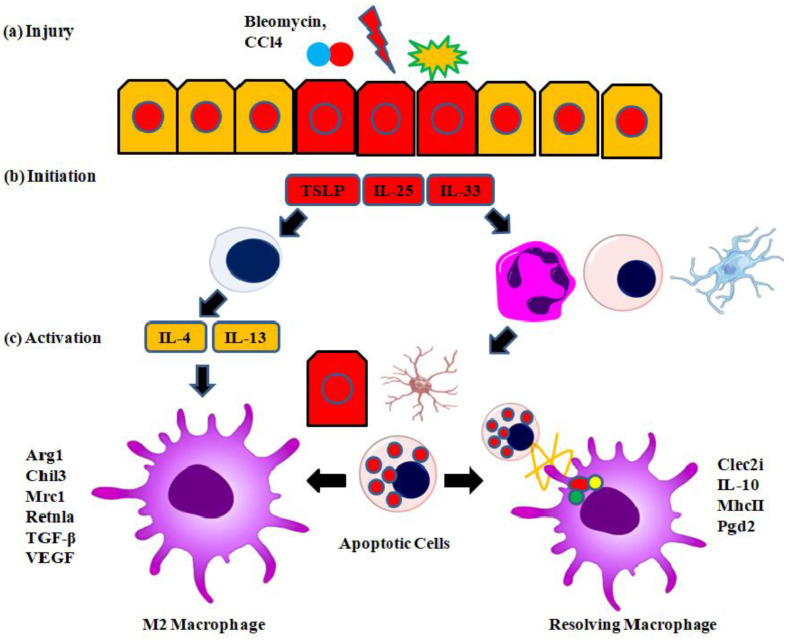
Stimulation of wound-healing macrophages. (**a**) A break in the barrier results from burn injuries, helminth infections, chemical injuries (CCl4 and bleomycin), or wounds. (**b**) Dying cells produce cytokines (TSLP, IL-25, and IL-33) that stimulate Th2 cytokine (IL-4/IL-13)-producing cells, therefore starting the wound-healing response. Moreover, innate immune cells like neutrophils are called upon to eliminate invasive infections and then undergo apoptosis when the threat has been overcome. (**c**) Th2 cytokines stimulate M2 macrophages (**left**). Phosphocytosis of the apoptotic cells arising from the inflammation activates resolving macrophages (**right**), which are equally significant. Both Th2 cytokines and apoptotic cells impact the activation of macrophages, although M2 and resolving macrophages do not constitute discrete subsets; rather, they represent a continuum. TSLP = thymic stromal lymphopoietin (figure created by Biorender).

Breaks in the mucosal barrier result from burn injuries, helminth infections, chemical injuries (CCl4 and bleomycin), or wounds. Herein, the dying cells produce cytokines (TSLP, IL-25, and IL-33) that stimulate Th2 cytokine (IL-4/IL-13)-producing cells, therefore starting the wound-healing response. Moreover, innate immune cells like neutrophils are called upon to eliminate invasive infections and then undergo apoptosis when the threat has been overcome. Th2 cytokines stimulate M2 macrophages (left). Phosphocytosis of the apoptotic cells arising from the inflammation activates resolving macrophages (right), which are equally significant. Both Th2 cytokines and apoptotic cells impact the activation of macrophages, although M2 and resolving macrophages do not constitute discrete subsets; rather, they represent a continuum. Thymic stromal lymphopoietin, or TSLP, is shown in Figure 5. Macrophage activation is guided by many soluble and cellular cues throughout the remodeling of tissue, and a reduction in inflammation is the last phase of wound healing. Apoptotic cells that produce an anti-inflammatory macrophage phenotype and Th2 cytokines that start a tissue remodeling “M2” program are two examples of these components. We will review the essential elements of various activation programs and their interplay to promote optimal wound healing. We have also discussed some newly found factors that influence these activation programs of macrophages (Figure 5) [59,60,61].

### 6.2. Macrophage Modulators and Promoters of Fibrosis and Wound Healing

**Figure 6:**
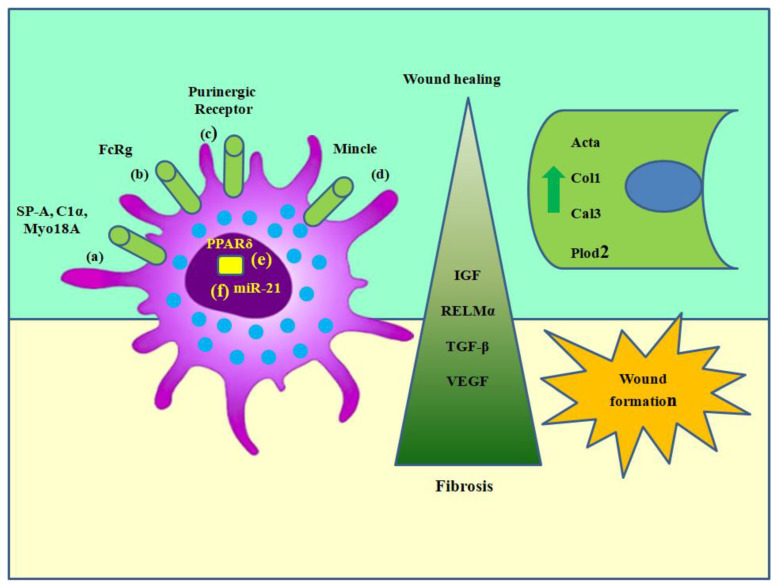
Macrophage modulators and promoters of fibrosis and wound healing. The following surface markers increase the activation of wound-healing macrophages: (a) Myo18A receptor signaling; (b) immune complexes that mediate FccR-mediated signaling; (c) ATP or adenosine binding to purinergic receptors; (d) expression of Mincle on the surface and intracellular factors; (e) nuclear receptor PPARc; and (f) micro-RNA 21. These improve the ability of macrophage effectors to aid in wound healing, but if taken in excess, they might cause fibrosis. ATP = adenosine triphosphate (figure created by BioRender).

The following surface markers increase the activation of wound-healing macrophages: Myo18A receptor signaling; immune complexes that mediate FccR-mediated signaling; ATP or adenosine binding to purinergic receptors; expression of macrophage-inducible Ca²⁺-dependent lectin receptors (also known as Mincle) on the surface and intracellular factors; nuclear receptor PPARc; and micro-RNA 21. These improve the ability of macrophage effectors to aid in wound healing, but if taken in excess, they might cause fibrosis (Figure 6).

Inadequate wound healing is another serious and sometimes lethal effect of diabetes. Although there are many contributing factors, defective macrophage responses are one of the ways via which diabetic patients experience chronic wounds that do not heal [84,85,86,87,88]. Specifically, diabetes affects the nuclear receptor PPARc’s ability to activate macrophages. By upregulating wound-healing genes and downregulating pro-inflammatory cytokine production, PPARc activation facilitates wound repair. The increase in PPARc activity also results in the development of tissue for granulation, blood vessel development, and collagen deposition, all of which are necessary for wound healing [89,90,91,92,93]. Reduced PPARc activity in diabetic wounds was caused by the ongoing manufacture of IL-1b, which activated the inflammasome [94,95,96,97,98]. One potential treatment approach to enhance wound-healing macrophages is by treating the lesion with PPARc agonists, which may reverse the condition [99,100,101]. Furthermore, it was shown that Mincle, a C-type lectin synthesized on macrophages, has a role in mediating fibrosis. The consumption of a meal rich in fat led to an elevation in the production of Mincle in macrophages located in the crown-like structures of the epididymal fat, which is a distinctive feature of adipose tissue in individuals who are obese. Compared to CD11b + F4/80hi cells, CD11b + F4/80lo cells transcribed Mincle over-predominantly. These macrophages that expressed Mincle exhibited reduced CD206 expression and increased CD11c expression, which is consistent with other research indicating that Mincle is only expressed by classically activated macrophages. Mice lacking a-SMA+ cells and myofibroblasts, as well as those with less interstitial fibrosis in the epididymal adipose tissue, were protected against insulin resistance and hepatic steatosis [102,103,104].

**Figure 7:**
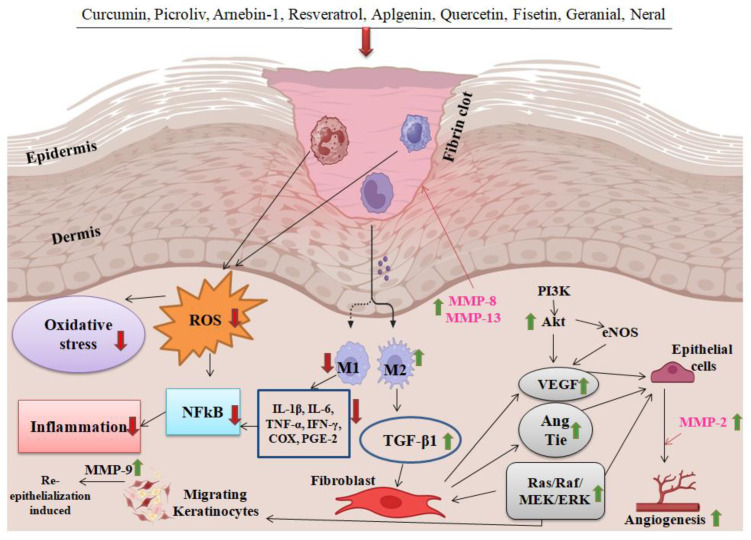
Mechanism of macrophage activation and wound resolution by natural bioactive compounds and phytochemicals in animal tissue. Different types of phytochemicals promote wound healing by erythrocyte stimulation near the ulcer wound. Fibrin clots promote the downregulation of oxidative stress, ROS generation, inflammation and translocation of NF-kβ, and release of inflammatory cytokines and induce the proliferation of keratinocytes and fibroblasts. The accumulated fibroblasts stimulate TGF-β, VEGF, angiotensin, and MAPK pathways, which promote the release of MMPs following angiogenesis, which promotes the acceleration of the wound-healing process (figure created by Biorender). Bold red downward arrow indicates inhibition or downregulation and bold green upward arrow indicates activation or upregulation. Black doted arrow indicate differentiation.

The timing of M2 macrophage administration may have a significant impact on the pathogenic or favorable effects on fibrosis [105,106,107,108,109,110]. Weng et al. investigated the function of M2 macrophages in a liver fibrosis model with spontaneous recovery. In this model, mice were exposed to CCL4 for an extended period, and then the liver was left to heal naturally without any further treatment. Remarkably, animals lacking IL-4Ra, specifically in their macrophages, were protected against the progression of hepatic fibrosis after CCl4 treatment. However, their recovery phase exhibited a delayed reversal of fibrosis. Using an antisense IL-4Ra nucleotide at various time points, the phase-specific function of M2 macrophages was verified. It was observed that M2 macrophage activation (Figure 7) early on increases fibrosis, whereas M2 macrophage activation later on speeds up fibrosis reversal [111,112]. 

### 6.3. Phytochemicals in Macrophage Activation for Wound Healing

Multiple growth factors, cytokines, and chemokines collaborate to facilitate the process of wound healing. The wound environment and careful administration of the TGF-β and VEGF families facilitate this process by enhancing their activity [112]. By concurrently raising the levels of anti-inflammatory mediators, most notably IL-10, and decreasing the levels of inflammatory mediators, including PGE2, LTB-4, IL-1β, TNF-α, IL-6, IFN-γ, and COX, flavonoids exhibit strong anti-inflammatory properties. Effectively suppressing the M1 phenotype, quercetin promotes M2-type cells and amplifies inflammatory signals [113]. Nevertheless, it cannot be said that all flavonoids regulate this macrophage transformation. Research indicates that the flavonoid glycoside vaccarin may stimulate the production of CD31 and increase the protein expression of p-Akt and p-Erk [114]. This can lead to the formation of new blood vessels and accelerate the process of wound healing. These findings indicate that flavonoid glycosides are likely the main active components in wound healing. Alongside other flavonoids and chlorogenic acids, they may enhance the therapeutic effects on joints by promoting the growth of beneficial substances, speeding up the formation of new blood vessels, and stimulating tissue development at different stages [115].

Catechins (flavan-3-ol) are the most extensively studied flavonoids due to their impact on wound healing [116]. Many studies have proposed that flavonoids like apigenin might help cure skin injuries by preventing fibroblast growth since excessive or inadequate fibroblast activity hinders wound healing [117]. Dietary flavonoids like lutein are often present in common fruits and vegetables, as well as a number of medicinal herbs. It has been used in several wound models as a wound healer. Many medicinal herbs contain rutin, also known as quercetin-3-O-rutinoside, which has the ability to speed up the healing of wounds [118]. 

Strong inhibitors of reactive oxygen species (ROS), which are essential constituents of diets high in antioxidants, have been found in many flavonoids. An investigation was conducted to examine the impact of reactive oxygen species (ROS) on the oxidation process of quercetin, kaempferol, morin, catechin, and naringenin. There were notable differences in the reaction rates measured by oxygen consumption and spectrophotometry. Because of its strong anti-inflammatory and antioxidant qualities, quercetin is a good choice to speed up the healing of wounds [114]. 

Previous studies have shown that flavonoids are effective in reducing the time it takes for wounds to heal by regulating MMP-2 activity and collagen breakdown after a 24 h course of treatment [119]. After receiving quercetin-3-oleate at its maximum concentration of 1 μM, there was a 51% increase in the pace of wound healing along with a little synthesis of TGF-β and release of MMP-9 [120]. The lack of MMP-9 in HaCaT cell cultures implies that other signaling pathways may control these cells’ ability to repair wounds, even when TGF-β overexpression was strongly stimulated. Thus, studies were conducted to determine hesperidin’s (a flavone glycoside) impact on diabetic foot ulcers [121]. In the case of a long-lasting diabetic foot ulcer, the speed at which the lesion healed was slower than 21 days, unlike the mice in the control group. There has been a notable increase in wound closure, as well as acceleration in the formation of new blood vessels by increasing the synthesis of TGF-β and Smad-2/3 mRNA, as well as VEGF-c and Ang-1/Tie-2.

Angiogenesis is an essential process that, at the same time, supplies wounds with new cells and nutrients and encourages the formation of new tissue and organs [122]. Furthermore, flavonoids’ additional antibacterial qualities contribute to the pace at which they induce epithelialization. MMP-2 plays a vital role in modifying the matrix involved in angiogenesis, whereas MMP-9 induces the process of re-epithelization in the first phases of healing [123]. During the process of wound healing, MMP-8, also known as collagenase-2, breaks down collagens, specifically collagenase. On the other hand, MMP-13, or collagenase-3, indirectly promotes re-epithelialization by affecting wound contraction. This review successfully demonstrated the impact of flavonoids on MMPs [124]. Flavones and flavanols have the ability to enhance tissue regeneration by increasing the expression of MMPs 2, 8, 9, and 13. TGF-β is among the several cytokines and growth factors that stimulate the transcriptional activation of MMPs. Keratinocyte migration is crucial for efficient re-epithelialization. Recent evidence has shown the essential role of Smads in transducing TGF cell signaling. Smad 2 and 3 are molecules that regulate the cellular processes related to wound healing. They do this by functioning as latent nuclear transcriptional activators [125]. 

By increasing VEGF levels and promoting Tie 1, Tie 2, and Ang-1 migration, some flavonoids demonstrated angiogenic qualities [126]. As a result, a continuous vascular network was established, and the transport of oxygen to growing tissues was improved, which promoted efficient wound healing. It is well acknowledged that VEGF is the main molecule that controls vascular expansion during tissue regeneration and embryonic development [127]. The formation and flexibility of new blood vessels are facilitated by Tie receptors (1 and 2), which are mostly present in endothelial cells. The blood artery wall is entirely covered by Ang-1, which attaches to Tie 2 and maintains its structural integrity throughout the formation process [128]. 

The significance of the MAPK and PI3K/AKT signaling pathways in angiogenesis throughout the wound-healing process has previously been well established by the scientific literature. The main factor influencing cell proliferation is the Raf/MEK/Erk signaling pathway [129]. Nitric oxide (NO) is produced as a consequence of eNOS activation, which is brought on by stimulation of the PI3K/AKT/mTOR pathway. Angiogenesis, vascular remodeling, and vasodilation may then be brought on by this. In a model of full-thickness wounds, the review showed that icariin efficiently activated Akt and ERK. Skin wound healing was expedited as a result of this activation, which induced keratinocyte migration and proliferation (Figure 7). 

Burn wounds may be healed well by α-pinene, which is present in significant concentrations in *Pistacia atlantica* resin. This is explained by its capacity to stimulate angiogenesis and raise levels of platelet-derived growth factor (PDGF) and basic fibroblast growth factor (bFGF). Additionally, α and β-pinene, which are found in *Salvia officinalis* EO, have an anti-inflammatory effect in vitro by inhibiting the production of NO in mouse macrophages. Greater than the effects of α- and β-pinenes, sabinene and the α- and β-isoforms of citral have shown strong anti-inflammatory properties by preventing macrophages from producing NO [130]. Nonetheless, we postulate that the inflammatory response could be regulated by the wound-healing process. Evidence showing *Salvia officinalis* essential oil’s (EO) anti-inflammatory qualities when used topically to treat plantar edema in rats lends credence to this theory. Moreover, several components of it have been identified as anti-inflammatory agents that impede the synthesis of NO. Furthermore, several constituents increase the synthesis of essential chemicals, such as FGF and PDGF, which facilitate wound healing and stimulate angiogenesis and antioxidant activity [131]. This may provide a defense against the oxidative stress that arises during the period of inflammation. According to recent research, citral, geranial, neral, and α-phellandrene may stop leukocyte adhesion and rolling, and they can also stop the production of pro-inflammatory cytokines, including IL-6 and TNF-α [132,133]. Furthermore, they have the ability to prevent mast cell degranulation caused by compound 48/80. The natural chemicals found in rosehip oil enhanced the healing of scars by preventing the transition of cells from the epithelial to the mesenchymal state [134,135]. This resulted in an increase in the concentration of collagen III in the tissue of the wound and facilitated the transformation of macrophages from the M1 to M2 phenotype [136]. According to some authors, essential oils are said to have antibacterial qualities that may assist in the healing process of wounds. The presence of phenolic compounds, namely carvacrol and thymol, is responsible for the features shown by these substances [137]. A possible method of action is that the inherent compounds included in essential oils specifically interact with the phospholipids present in bacterial cell walls and cell membranes, hence enhancing permeability and finally causing cell lysis. Extracts from *Cymbopogon citratus*, *Angelica dahurica*, *Rheum officinale*, and *Euterpe oleracea* exhibited reduced plasma levels of TNF-α, TGF-β1, IL-6, and IL-1β as compared to the treatment groups. This finding indicates that these extracts promote the inflammatory stage of the chronic wound-healing process [138]. 

## 7. Phytonutrients in the Treatment of Diabetic Wounds with Clinical Trials in Rodent Models

Diabetic wound-related complications continue to be common despite recent advancements in medicine and surgery. Techniques used for wound care should remove infections from wounds while maintaining sufficient blood flow [139]. This lowers the danger of amputation, guarantees quick healing without infection spreading, and enhances quality of life [140,141,142,143,144,145].

**Figure 8:**
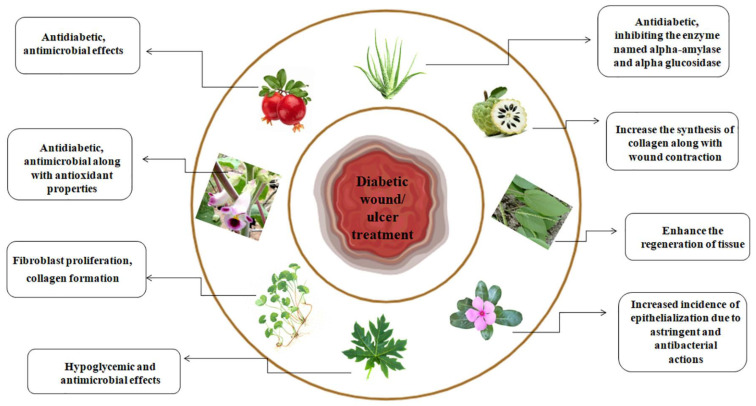
Effects of different phytochemicals on diabetic wounds/ulcers (figure created by MS Power Point 2007).

Tissue engineering techniques, including platelet-rich plasma, cytokine-inhibiting agents, recombinant growth regulators, MMP-inhibiting agents, skin replacements, stem cells, lasers, and stimulants of the extracellular matrix and blood vessel development, are currently the main focus of research on diabetic wounds [146]. Living skin substitutes include fibroblasts, keratinocytes, and stem cells. They can be utilized alone or in conjunction with extracellular matrices or growth factors. Even though diabetic people do not always experience complete wound closure without scarring, healing benefits, including lower infection risk and better quality of life after amputation, are still significant to these patients. In order to effectively manage diabetic wounds, therapeutic interventions should positively influence gene expression, decrease microbial invasion, and control inflammatory processes, oxidative stress, and blood glucose levels without changing any steps or phases involved in wound healing (Figure 8; Table 3) [147,148,149]. 

The use of medicinal plants for therapeutic purposes, or phytomedicine, has a long history in many cultures as well as in the current period. It has been found that these medicinal plants successfully boost the biological system with negligible or no adverse effects. Secondary metabolites are health-promoting chemicals found in herbal remedies, which are valuable due to their nutritional value and pharmacological qualities. These metabolites are crucial to the global healthcare system [150]. These natural compounds are better than manufactured ones since they are antioxidant-rich, which eliminates safety issues. 

Plants derived from many sources, such as ginseng, turmeric, and neem bark, are traditionally very important for wound healing. Among their bioactive compounds with therapeutic qualities are flavonoids, glycosides, steroids, mucilage, essential oils, saponins, and resins. They have a role in a number of wound-healing processes, such as enhanced lesion shrinkage, the accumulation of collagen, granulation tissue development, and inflammation management [151]. The application of natural product-centered therapy in plant-based medicines is widely praised worldwide. However, in order for these natural products to be taken into consideration for the treatment of diabetic wounds, they need to have certain qualities. Controlling hyperglycemia, physiologic inflammatory conditions, oxidative stress, and infection are a few of these, but they also include altering the expression of important proteins that are involved in the healing process of ulcers and wounds [152].

Immediate closure of the wound, minimal colonization by microbes, moderate growth elements, cytokine release, and regulated blood glucose levels are all indicators of a healing wound (Table 3). Recent findings overlooked all these factors that contribute to long-term, persistent inflammation. The prognosis for diabetic wounds is miserable, and little research has been conducted on useful therapies for managing them. Furthermore, a thorough understanding of the intricate mechanism underpinning effective healing is required. As a result, using medicinal plants alone or in combination to directly target these pathways may be a potential strategy to manage wounds caused by diabetes [153].

Numerous plant and herb species with the ability to cure wounds have been found in Africa and other developing nations as a result of ethnobotanical studies. Table 3 highlights numerous bioactive compounds of therapeutic herbs along with their impacts on diabetic wound healing. Medicinal herbs are used to treat and control wounds by debridement, decontamination, and creating an environment that is conducive to the body’s own healing mechanisms [154]. The use of medicinal plants’ bioactive compounds and phytochemicals in the treatment of wounds, whether from diabetes or not, has attracted attention recently because it is believed that these substances are less dangerous and have fewer side effects than traditional therapeutic agents. The implementation of medicinal plant-derived phytochemicals is one of the therapeutic approaches for the treatment of impaired healing of diabetic wounds, which is viewed by medical personnel worldwide as a serious health challenge due to non-specific etiology in certain cases. This is especially true in settings with limited resources [155].

*Catharanthus roseus* (*C. roseus*), often called *Vinca rosea*, is a shrub with distinctive purple or white flowers that originated in Madagascar. The abundance of several phytochemicals dispersed throughout the plant has been connected to the hypoglycemic character of the plants. Nayak examined the impact of *C. roseus* ethanol extract on diabetic rats’ ability to repair wounds [156]. The investigation was driven by the concern that existing methods of treating chronic diabetic wounds, including irrigation, corticosteroids, antibiotics, proteolytic enzymes, tissue transplants, and debridement, may have unintended consequences. Because of their astringent and antimicrobial properties, which might be responsible for wound contraction along with a boost in the degree of epithelialization, it is assumed that components of *C. roseus*, such as alkaloids, tannins, and triterpenoids, might have played a significant role in the diabetic rats’ wound-healing processes [157].

*Centella asiatica* or *Asiatic centella* (*C. asiatica*) is frequently utilized as a therapeutic herb in traditional Chinese, African, and Ayurvedic medicine. It has been demonstrated to increase collagen production and the growth of fibroblasts [158]. After the induction of diabetes, excision wounds were established on day 3 [159]. In the testing rats, incisions were made on the left side of the dorsal shoulder skin. After anesthesia, an excision wound was created by severing a 15 mm by 15 mm section of skin from an area that had been shaved. The study came to the conclusion that *C. asiatica* could help animals with diabetes recover their wounds; nevertheless, more research is advised to pinpoint the precise components causing the healing actions [160].

*Acalypha langiana* (*A. langiana*) is a wild herbaceous plant whose leaves are frequently utilized in conventional medical practices to treat infections caused by bacteria and wounds. The authors selected a liquid extract of fresh *A. langiana* leaves for this investigation. It just took a single administration of streptozotocin (STZ) to cause diabetes in the rats. Rats that displayed elevated blood glucose levels (>250 mg/dL) on the seventh day after being induced with diabetes were used for wounding, and two varieties of wounds (incision and excision) were created in the study rats. The diabetic rats’ wound healing was significantly and dose-dependently impacted by the topical administration of *A. langiana’*s aqueous leaf extract. More specifically, tensile strength was substantially improved in incision wounds addressed with the extracts (flavonoids). According to the findings of the study, tissue regrowth in the granulation tissue regions of the extract-treated group was significantly faster than in the control wounds [161]. 

*Aloe vera* (*A. vera*) has been acknowledged and utilized in conventional medicine to treat a wide range of medical issues in many cultures throughout the world. Its antimicrobial and antidiabetic properties have been demonstrated. An in vivo investigation was conducted using male Wistar rats to assess the plant extract’s ability to heal wounds. On day seven, following the use of streptozotocin (STZ) to induce diabetes, sores developed. *A. vera* extract was applied to the wounds for a set number of days, treating both excision and incision wounds. On clear paper, excisions were seen and quantified on a millimeter scale in order to determine the rate of wound shrinkage. With the help of the healed wound area%, the change in wound size was measured. An indicator of the epithelialization phase was the amount of time needed for the wound to completely epithelialize. The amount of collagen increased dramatically after the fourth day of granulation tissues. The granulation tissues of animals given plant extract also had higher concentrations of DNA and protein. The research finds that *A. vera* extract sped up the healing of diabetic wounds and suggests that *A. vera* treatment may be helpful for the various phases of wound healing, such as collagen formation and reduction, which led to faster healing than in untreated animals [162].

*Martynia annua* (*M. annua*) is an annual herb with glandular hairs that is mostly used to treat tuberculosis and epilepsy. Additionally, it is utilized to treat wounds, inflammation, and irritation of the throat [163]. The plant’s leaves were obtained, identified, and treated using the previously mentioned procedure [164]. After injecting streptozotocin to induce diabetes in Wistar rats, an excision wound was created. When contrasted with the control group, the wounds treated with the extract were shown to exhibit a substantial contraction. It was also shown that animals injected with plant extract had considerably greater amounts of hydroxyproline than the control group. Histological analysis revealed that the mice treated with *M. annua* extract had more fibroblast cells and well-organized collagen fibers [165].

*Punica granatum* (*P. granatum*) is a significant healing plant in the Middle East. The flowers of these plants are frequently used for wound treatment and are utilized as antimicrobial agents [166]. An experiment was conducted using male Wistar rats to examine the plant’s potential for wound healing. The skin wound treated with the extract-containing ointment base showed better tissue regeneration on day nine, according to the results. On day 18 of therapy, the diabetic animal showed signs of *P. granatum’*s ability to heal wounds. This finding may support the conventional healthcare practice of using *P. granatum* to treat wounds [167].

There have been studies that indicate antibacterial, antioxidant, and anti-inflammatory properties in *Carica papaya* (*C. papaya*) extract. In order to examine the ability of *C. papaya* to heal wounds in an animal model with diabetes, unripe papaya fruits were gathered from their local area and processed using the previously mentioned technique [168]. The antibacterial and proteolytic enzymatic properties of *C. papaya*, which are present in the plant’s main ingredients, chymopapain and papain, are linked to the fruit’s ability to heal wounds. The absence of biofilm in the diabetic rats treated with the extract indicates that the enzyme components of *C. papaya* were able to compromise the protective layers of biofilm against oxygenation and UV light, hence promoting bacterial imbalance [169].

*Annona squamosa (A. squamosa)* is another name for custard apple. It is grown in India, where the natives utilize the seeds and foliage to treat diabetes as well as additional illnesses like fever and ulcers [170]. The effectiveness of the ethanolic extract of the plant’s leaves on wound healing in streptozotocin (STZ)-induced diabetic rats was evaluated by Ponrasu and Suguna [171]. This paper shows that *A. squamosa* therapy considerably improved overall excision and incision wounds. Its high phenolic content has been linked to its ability to promote collagen production and wound contraction, which in turn is thought to be responsible for its wound-healing activity [171,172,173,174,175,176,177] (Table 4). Integrating therapeutic substances like growth-promoting agents, nitric oxide, nucleic acid, antioxidants, and antimicrobial agents into injured tissue has been scientifically proven to effectively stimulate cell growth and migration, new blood vessel formation, and collagen secretion [178,179,180]. Additionally, it might hinder the development of dangerous germs, which can hinder the healing of chronic wounds [181,182,183]. Due to their structural similarity to the extracellular matrix seen in nature, nanofibers have attracted a lot of interest [184,185]. The positive attributes of nanofibers, including their huge surface-area-to-volume ratio, improved porosity, tunable mechanical properties, and capacity to store nanoparticles and bioactive compounds for controlled release, aid in the healing of wounds [186,187,188,189,190,191,192,193]. This is because they allow cells and the matrix to actively engage throughout the functionalization and remodeling processes but require extensive investigation for future endeavors. Hydrogels are three-dimensional polymer networks with a high affinity for water; research has shown their effectiveness in tissue engineering and drug delivery [194,195,196].

### Clinical Trials in Humans

While a large body of research has been conducted on animals, very few clinical studies have been carried out to clarify the impact of herbal medications on the healing process of diabetic wounds. Unfortunately, no human research has been conducted to determine how active ingredients made from herbal remedies affect diabetic wound healing [197]. The long duration, high expense, and specialized regulatory processes associated with clinical studies intended to demonstrate the safety and effectiveness of herbal treatments account for the disparity between research conducted on animals and humans. Moreover, regulatory bodies must provide individual permission. Within the scientific community, research is still being conducted in spite of these restrictions. Diabetic foot ulcer (DFU) models serve as the basis for the investigations conducted on humans. The chronic nature of non-healing wounds, neuropathy, and peripheral vascular disease are all strongly correlated with the DFU model. Over time, increased blood glucose causes nerve fibers to eventually break down and capillaries to shrink. This accelerates the development of vasoconstriction and atherosclerosis, which ultimately results in the development of occlusive artery disease and DFUs [198]. Diabetic foot ulcers, or DFUs, may also lead to serious effects, including the need for amputation, and are a major source of disease. This is particularly true if the DFUs eventually become infected. Multidisciplinary approaches are sometimes required for the best management of certain diabetic foot issues. In order to implement this technique, wound debridement, pressure relief, blood sugar regulation, negative pressure therapy, and surgical procedures are often used. These tactics simultaneously help to remove excess exudates and microbes from the wound bed and provide the best possible environment for tissue regeneration. More research is required on phytochemical applications, but when *Aloe vera*, olive oil, kiwifruit, and *Securinega leucopyrus* were applied topically, foot ulcers either completely healed or shrank in size [199]. When hydrocolloid fiber dressings were used as the control, there were no appreciable changes in the size of the wounds after the application of a cream containing the active ingredient isolated from *Plectranthus amboinicus* and *Centella asiatica*.

## 8. Meta-Analysis, Systematic Analysis, and Statistical Data on Diabetic Wound Healing

An emerging technique that enables the simultaneous assessment of several treatment options—some of which may not have been investigated in the initial studies—is meta-analysis. Randomized control trial (RCT) data are used in the bulk of network meta-analyses. Randomized studies are seldom taken into consideration. RCTs may be enhanced by non-randomized research (Table 5). Problems include issues with ethics, constrained participant selection, tiny sample sizes, and infrequent follow-ups. In a previous study, re-epithelialization rate = 5.06 (95% CI = 3.75–6.37; *p* < 0.00001); SMD = 5.42 (95% CI = 4.40–6.44; *p* < 0.00001); neovascular density = 5.48 (95% CI = 4.31–6.64; *p* < 0.00001); and collagen deposition = 4.78 (95% CI = 3.58–5.98; *p* < 0.0001) [200]. Furthermore, exosome therapy markedly decreased the expression of inflammatory factors. A statistical study revealed that every measurement of a wound had a linear healing slope larger than R = 0.70 and *p* = 0.0001. This implies that prognostic indications for wound healing might be derived from all five wound features [201]. Comparing the insulin-treated group to the control group, the mean improvement in wound healing (mm^2^ /day) was statistically significant (IV = 11.84; 95% CI: 0.64–23.04; *p* = 0.04; I2 = 97%). The secondary results demonstrated that there is no statistically significant difference in the wound-healing time (days) (IV = −5.40; 95% CI: −11.28 to 0.48; *p* = 0.07; I2 = 89%), that the insulin group has a significant reduction in wound area, that localized insulin has no adverse effects, and that quality of life improves as the wound heals [202]. A statistical analysis revealed that diabetes patients’ wound healing after TTA was influenced by their age (*p* = 0.007). It was found that wound severity was not substantially affected by comorbidities other than diabetes (*p* =0.209), gender (*p* = 0.677), preoperative anemia (*p* = 0.102), intraoperative blood transfusion (*p* = 0.633), antithrombotic or anticoagulant drugs (*p* = 0.556), PTA or bypass surgery (*p* = 0.6) [203]. This research found that age had a substantial impact on wound healing in individuals with diabetes undergoing TTA. 

## 9. Conclusions

Wound healing involves hemostasis, inflammation, proliferation, and remodeling to restore devitalized cellular structures. Natural wound-healing treatments are accessible and inexpensive, making them advantageous for patients. Plant medicine has existed for centuries in many cultures and is being used today. With little side effects, these therapeutic herbs enhance the biological system. Secondary metabolites, nutritional and pharmacological compounds in herbal treatments, promote health. Indian spice-derived polyphenol reduces pro-inflammatory cytokines, including TNF-α, IL-1β, and IL-6, via inhibiting the NF-κβ pathway. Curcumin modulates inflammation and wound healing via binding to TLRs and modulating downstream signaling pathways such as NF-κβ, MAPK, and AP-1. This paper reviewed natural products, bioactive compounds, and secondary metabolites for wound healing based on plant and animal bioactivities, offering an overview of the chemical origin of natural products and biological wound-healing processes. Macrophages are essential to wound healing and a worthwhile therapeutic target. Malfunctioning macrophages contribute to slower healing of wounds in the context of aging and diabetes, as well as to excessive tissue regeneration in fibrosis. Increasing data suggest that macrophages are highly adaptable, have a long lifespan, and may modify their characteristics in anticipation of external signals. Thus, they may alter their function in aberrant wounds to improve results. Even though activator mechanisms along with downstream agents of wound-healing macrophages are well characterized, macrophage-specific wound-healing techniques have problems. 

Optimal healing of a cutaneous wound necessitates a coordinated integration of the intricate biological and molecular processes of cell migration, proliferation, and extracellular matrix deposition and remodeling. Cellular responses to inflammatory mediators, growth factors, cytokines, and mechanical stresses must be accurate and specific. Nonetheless, this systematic advancement of the healing process is disrupted in chronic wounds, particularly those resulting from diabetes. Various pathogenic anomalies, including disease-specific intrinsic deficiencies in blood supply, angiogenesis, and matrix turnover, as well as extrinsic variables stemming from infection and persistent stress, contribute to impaired healing. Nevertheless, in spite of these challenges, there is growing reason for optimism about the treatment of diabetic and other chronic wounds. Improved comprehension and rectification of pathogenic causes, along with greater compliance to care standards and technical advancements in biological agents, are instilling renewed optimism for delayed healing.

## Figures and Tables

**Table 1:** List of herbal plants with bioactive compounds and phytochemicals with their pharmacological significances.

Sl. No.	Name of Herbal Plants	Parts Use	Phytochemicals	Traditional Application and Pharmacological Impacts	Refs.
1.	*Rosmarinus officinalis*	Aerial parts	Alkaloids, flavonoids	Diabetic wound treatment, antimicrobial, anti-inflammatory, and antioxidant activities	[2]
2.	*Carica papaya*	Unripe fruits	Terpenoids, phenolics, and alkaloids	Wound healing, antimicrobial, antioxidant, and anti-inflammatory activities	[9,11,12]
3.	*Radix rehmanniae*	Leaves	Tannins, saponins, xanthones, and alkaloids	Diabetic foot ulcer healing, tissue regeneration, angiogenesis, and inflammation control, antimicrobial, anti-cancer, antioxidant	[8]
4.	*Annona squamosa*	Leaves	Glycosides, phenolic, alkaloids	Diabetic ulcer treatment, fever treatment, anti-inflammatory, anti-cancer, anti-allergic, antiviral, and antioxidative	[13,14]
5.	*Azadirachta indica*	Leaves	Flavonoid rutin like quercetin and tetranortriterpenoid meliacinolin, glycocides	Diabetic wounds, anti-inflammatory, anti-cancer, anti-allergic, antiviral, and antioxidative	[15,16]
6.	*Annona squamosa*	Leaves	Flavonoids like quercetin and isoquercetin, glycocides	Diabetic wounds	[17,18]
7.	*Abrus precatorius*	Leaves, seeds	Flavonoids, triterpene glycosides, abrin, and alkaloids	Diabetes, wounds, tetanus fever, cold, cough	[7]
8.	*Acacia arabica*	Bark, roots	Alkaloids, flavonoids, and glycosides	Diabetes, wound, diarrhea, diuretic, liver tonic	[19]
9.	*Catharanthus roseus*	Leaves	Flavonoids and alkaloids	Chronic diabetic wounds	[20]
10.	*Centella asiatica*	Leaves	Alkaloids, flavonoids, and glycosides	Diabetic dermal wound healing, antibacterial, antioxidant, and anti-inflammatory activities	[21]
11.	*Acalypha langiana*	Fresh leaves	Flavonoids, triterpene, and glycosides	Diabetic wound healing, antibacterial, increased tissue regeneration	[22]
12.	*Hylocereus undatus*	Leaves, flowers, and fruits	Flavonoids, alkaloids, saponins, and steroids	Antibacterial, increased tensile strength	[23]
13.	*Punica granatum*	Flowers	Polyphenolic compounds found in *P. granatum* include pomegranatate, ellagic acid, ethyl brevifolincarboxylate, and maslinic and urolic acids, as well as daucostero.	Astringent and hemostatic agent, possessing antibacterial, antifungal, and antiviral properties. Utilized as a remedy for cut wounds, diarrhea, and digestive issues.	[24]
14.	*Aloe vera*	Leaves	Flavonoids, anthraquinones, steroids, tannins, chromones, alkaloids, anthrones, and phenols	Wound-healing activity, antidiabetic, anti-inflammatory, and antibacterial activities	[6]
15.	*Martynia annua*	Flowers	Anthocyanin, niacin malvin, malvaline malvidin 3-(6î-malonylglucoside)-5-glucoside, and folic acid	Agents with antibacterial, antifungal, and antiviral properties are commonly employed in the management of wounds.	[24]

**Table 2:** List of phytochemicals and their mechanism of action in inflammatory pathways.

Phytochemical	Source	Mechanisms of Action	Affects	Refs.
Curcumin	*Curcuma longa*	- Blocks NF-κB pathway - Regulates TLRs, MAPK, and AP-1 - PPARγ inhibits the activity of NF-κB - Regulates the JAK/STAT pathway - Prevents NLRP3 inflammasome construction and activation	- Anti-inflammatory - Antioxidant - Antimicrobial - Angiogenic - Enhances wound healing	[25,26,27,28,29,30,31,32]
Picroliv	*Picrorhiza kurroa*	- Improves angiogenesis - Promotes re-epithelialization and neovascularization - Increases VEGF and insulin-like growth factor production	- Enhances wound healing	[25,26,27,28,29,30,31,32]
Arnebin-1	*Arnebia nobilis*	Traditional use in wound healing	- Enhances wound healing	[40]

**Table 3:** Some medicinal plants with their active ingredients for diabetic wound treatment.

Medicinal Plants with Family	Utilized Segments	Type of Extractions	Phytochemicals/Bioactive Compounds	Effects
*Aloe vera* (Asphodelaceae)	Leaves	Methanolic extracts	Polysaccharide	Antidiabetic, inhibiting the enzymes named alpha-amylase and alpha-glucosidase
*Annona squamosa* (Annonaceae)	Seeds, foliage	Ethanolic extracts	Phenols	Increase the synthesis of collagen along with wound contraction
*Acalypha langiana* *(Euphorbiaceae)*	Leaves	Aqueous extracts	Flavonoids	Enhance the regeneration of tissue
*Catharanthus roseus* (Apocynaceae)	Leaves	Ethanolic extracts	Alkaloids, tannins, triterpenoids	Increased incidence of epithelialization due to astringent and antibacterial actions
*Carica papaya* (Caricaceae)	Leaves	Ethanolic extracts	Flavonoids, alkaloids, glycoside, phenols	Hypoglycemic and antimicrobial effects
*Centella asiatica * (Apiaceae)	Foliage	Ethanolic extracts	Polyphenolic compounds	Fibroblast proliferation, collagen formation
*Martynia annua* (Martyniaceae)	Whole plants	Methanolic extracts	Tannins, terpenoids, flavonoids, glycosides, phenolic compounds	Antidiabetic, antimicrobial, and antioxidant properties
*Punica granatum * (Punicaceae)	Leaves	Methanolic extracts	Flavonoids	Antidiabetic, antimicrobial effects

**Table 4:** Chief bioactive compounds and their source, mechanism of action, and route of administration in rodent models.

Plant Source	Main Bioactive Components	Biological Activity	Mechanism of Action	In vivo Wound Model, Doses and Routes of Administration
*Achillea millefolium*	Flavonoids (chlorogenic acid, apigenin, artemetin, luteolin, quercetina and shaftoside)	Antibacterial Anti-inflammatory Re-epithelialization process	Modulates inflammatory cytokines and growth factors, activates Akt signaling pathways, stimulates collagen expression, stimulates keratinocyte differentiation and motility, reduces inflammatory mediators NO and PGE2.	Full-thickness incisional wound in Sprague Dawley rats—topical: 3% aqueous extract (AAE).
*Aloe vera*	Flavonoids (aloin, aleosin, emodin, rhein) Polysaccharides (acemannan, acetylated polymannan, and glucomannan)	Antibacterial Anti-inflammatory Re-epithelialization process	Modulates the inflammatory response, modulates signaling protein phosphorylation, stimulates collagen deposition and angiogenesis, strongly promotes fibroblast proliferation, and moderately stimulates keratinocyte migration.	Full-thickness wound in Wistar rats—topical: 25–50 mg/mL in gel. Full-thickness wounds in hairless mice—topical: 0.1% and 0.5% *w*/*w*. Full-thickness wounds in mice—topical: 10 and 50 mg/kg. Burn wounds in BALB/c mice—topical: aloe-emodin 12% *w*/*w*.
*Bletilla striata*	Flavonoids (anthocyanins) Polysaccharides (glucomannan) Triterpenoids Stilbenoids (bibenzyl, bletilol D, bletilol E, dihydrophenanthrene, and phenanthrene)	Antimicrobial and antiviral Antioxidative Anti-aging Anti-inflammatory Re-epithelialization process Hemostatic activity	Promotes expression of mediators of the inflammatory response (TNF-α, IL-1β, and IFN-γ); increases NO and promotes neutrophils, monocytes, and macrophages chemotaxis; promotes epithelial cell growth and fibroblast proliferation.	Partial-thickness burn wound model in mice—topical: 1 mg/mL BSP extract or BSP polysaccharide residue extract or mix
*Calendula officinalis*	Triterpenoids Flavonoids (rosmarinic acid, caffeic acid, 5-O-caffeoylquinic acid, isorhamnetin-3-oglucoside, isorhamnetin-3-orutinoside, kaempferol-3-orutinoside, quercetin-3-oglucoside, and quercetin-3-orutinoside) Coumarines Quinones	Anti-inflammatory Re-epithelialization process	Promotes expression of mediators of the inflammatory response; increases keratinocyte and fibroblast proliferation; stimulates collagen production and angiogenesis; inhibits lipoxygenase activity; reduces glutathione levels.	Full-thickness excisional wound in BALB/c—topical: 150 mg/kg BW ethanolic or water extract ointment. Metallic punch Wistar rats—topical: 100 μL of aqueous solution of 1% ethanolic extract. Incisional wound in Sprague Dawley rats—topical: 5–10% gel. Full-thickness wound in Wistar rats—topical: wound dressing in nanofibers with 2% *Calendula officinalis* extract.
*Casearia sylvestris*	Triterpenoids (clerodane diterpenes) Phenolic acids	Anti-inflammatory	Reduces early and late edema; reduces myeloperoxidase activity.	Full-thickness lesions—topical: 0.1, 0.3, 1.0 mg/site extract. Second-degree burns in Wistar rats—topical: biofilm with 1 g of lyophilized extract or spray with extract.
*Crocus sativus*	Carotenoids (crocin, crocetin, picrocrocin and safranal) Monoterpenoids Flavonoids (kempherol and quercetin) Phenolic acids	Fibroblasts from newborn mice: hydrogel with 160 mg/L crocin from saffron. Human dermal fibroblasts: 3.12–50 g/mL for 6–24 h C2C12, MCF7, HCT116 cell lines: 125 ug/mL of saffron anther extract	Reduces the level of pro-inflammatory cytokines (TNF-α and IL-6); increases level of anti-inflammatory cytokines (IL-4 and IL-10); inhibits lipid peroxidation; enhances vascularization; increases fibroblast proliferation.	Second-degree burns in Wistar rats—topical: cream with 20% pollen saffron. Full-thickness wound in Sprague Dawley rats—topical: pomade with 20% saffron extract
*Curcuma longa*	Curcuminoids (bisdemethoxycurcumin, curcumin, and demethoxycurcumin)	Antioxidant Radical-scavenging Anti-inflammatory Re-epithelialization process	Regulates many genes implicated in the initiation of inflammatory responses (NF-ƙB, AKT, PI3K, IKK); enhances fibroblast migration, granulation tissue formation, collagen deposition; increases TGF-β production; increases fibroblast proliferation.	Full-thickness wound in Balb/c mice—topical: gel 3% curcumin. Full-thickness wound in Wistar rats—topical: PCL nanofibers, 10% curcumin. Full-thickness wound in Wistar rats—topical: PVA nanofibers, 1% curcumin. Full-thickness wound model in SD rats—topical: 100–200 μg/mL curcumin nanoparticle-loaded dermal patch
*Glycyrrhiza glabra*	Flavonoids Terpenoids (glycyrrhizic acid, saponins, and triterpene) Chalcones (glycyglabrone and licochalcone C)	Antimicrobial Anti-inflammatory Antioxidant Re-epithelialization process	Increases collagen deposition; increases the wound-healing rate; reduces superoxide anions; inhibits NO production; increases fibroblast proliferation.	Sprague Dawley rat wounds—topical: 3% extract in cream. Guinea pig full-thickness wound—topical: 5% and 10% extract in cream.
*Malva sylvestris*	Polysaccharides Flavonoids (malvidin, malvin, delphinidin, genistein, myricetin, apigenin, quercetin, and kaempferol) Terpenoids (monoterpenes, diterpenes, sesquiterpenes, and norterpenes)	Antibacterial Antioxidant Anti-aging Anti-inflammatory	Modulates the inflammatory response; increases collagen deposition; Enhances vascularization; increases the wound-healing rate.	BALB/c mice cut wound—topical: 1% extract in cream. Second-degree burn wounds in rats—topical: 1–5–10% extract in cream. Diabetic streptozotocin-induced wound in Wistar rats—topical: 5–20% extract containing nanofibers
*Plantago L.*	Monoterpenoids (aucubin, acteoside, calceorioside B, catalpol, homoplantaginin, and plantamajoside)	Antibacterial Antioxidant Anti-inflammatory	Inhibits NO production; reduces superoxide anions; reduces pro-inflammatory cytochine levels (PGE2, TNF-α); decreases fibroblasts H_2_O_2_ cytotoxicity.	
*Salvia officinalis*	Terpenes (1,8-cineole) Oxysesquiterpenes (camphor, nonacosane, and pentacosane viridiflorol)	Anti-inflammatory Antimicrobial Antioxidant	Reduces pro-inflammatory cytokines; downregulates mRNA expression levels of IL-6, IL-1β, and TNF-α; augments fibroblast proliferation via enhancing cyclin-D1 expression.	BALB/c mouse excisional splinting model—topical: 0.5% *w/w* dry extract in cream. BALB/c mouse full-thickness wounds—topical: 2% and 4% essential oil ointment. Wistar rat wound models—topical: 1%, 3%, and 5% hydroalcoholic extract. Excision on streptozotocin-induced diabetic rats—topical: 0.5% and 1% essential oil.
*Rosmarinus officinalis*	Flavonoids (diosmin, eriocitrin, genkwanin isoscutellarein 7-O-glucoside, hispidulin 7-oglucosidehesperidin, and luteolin 3-o-β-D-glucuronide)	Antimicrobial Antioxidant Anti-inflammatory	Inhibits NO production; reduces inflammatory cytokine expression (IL-1β, IL-6, TNF-α); reduces expression of iNOS, COX-2, PIB and NFkβ/p65.	Full-thickness excision cutaneous wounds in alloxan-induced diabetic BALB/c mice—topical: 100% essential oil—intraperitoneal injection: 0.2 mL, 10% (*v/v*). Excision on streptozotocin-induced diabetic rats—topical: 100% essential oil. Full-thickness excision wound in Sprague Dawley rats—topical: 10% rosemary essential oil in chitosan.

**Table 5:** Summary of different types of studies with their objectives and outcomes related to diabetic wound healing.

Types of Study	Objective	Key Outcomes	Refs.
Meta-analysis	Mechanism of ROS in diabetic ulcer wounds.	Origin of diabetes mellitus	[1,2,3,4,5]
	Types of various pharmaceutical compounds in diabetic wound healing.	Mechanism of diabetic wound healing by various pharmaceutical compounds	[6,8,9,12,16,17,19,20,21,22,23,24]
	Phytochemicals regulate the human immune system and metabolic pathways.	Role of interleukin, cytokines, transcription factors	[25,26,27,28,29,30,31]
	Regulation of phytochemicals in AP-1, NF-kβ, ERK1/2-MAPK, PPAR gamma, JAK-STAT pathway.	Role and regulation of signaling pathways	[25,32,40]
Systematic review	To create solutions that address elements of cell biology and wound biochemistry related to chronic wound healing.	There are several challenges in this topic when analyzing the data, especially since there are few controlled studies and most of them have low methodological quality.	[200]
	Noninvasive screening assessments for predicting wound healing and the risk of amputation in diabetic foot ulcers.	Numerous assessments may forecast wound healing in diabetic foot ulcers; yet, the majority of existing information focuses only on transcutaneous oxygen measurement and the ankle–brachial index. The quality of the evidence is inadequate, necessitating more study to provide superior comparative effectiveness data.	[201]
	To evaluate the impairment of wound healing in diabetic mice models and to assess the quality of previous studies.	Numerous rodent models of diabetes may mimic poor wound healing; however, these models still need to be refined to make them more clinically relevant.	[202]
	There are now many techniques for the debridement of diabetic foot ulcers. It is unclear how effective any of these approaches is in comparison.	Due to methodologic constraints and imprecision, comparative efficacy evidence between some approaches and supporting data for others is poor. Thus, current debridement methods should be based on competence, patient preferences, clinical context, and cost.	[203]
	To examine the relative benefits of hydrogel dressings against traditional dressings in the management of diabetic foot ulcers.	The meta-analysis demonstrated that hydrogel dressings are superior to traditional dressings in the treatment of diabetic foot ulcers (DFUs).	[204]
Statistical analysis	To evaluate the efficacy of exosomes in the treatment of diabetic wounds.	In terms of wound-healing rate (SMD = 5.42; 95% CI = 4.40–6.44; *p* < 0.00001), neovascular density (SMD = 5.48; 95% CI = 4.31–6.64; *p* < 0.00001), re-epithelialization rate (SMD = 5.06; 95% CI = 3.75–6.37; *p* < 0.00001), collagen deposition (SMD = 4.78; 95% CI = 3.58–5.98; *p* < 0.00001) were all shown to be superior to control therapy in pooled analyses. Furthermore, the exosome therapy group showed a considerable downregulation of inflammatory factor expression.	[205]
	More methods for measuring wounds are now available to doctors because of 3D wound imaging. There is currently no information available to help physicians determine which 3D measures might provide the most accurate indicator of a wound’s ability to heal.	Each wound measurement showed a linear healing slope with a value larger than R 0.70 and a statistical significance of *p* = 0.0001, according to statistical analysis. This shows that each of the five wound parameters may be used as a helpful prognostic indicator while the wound heals.	[206]
	To investigate how localized insulin injection affects individuals with diabetes’ wound healing and its safety.	The study’s primary outcome examined the rate of wound healing (mm^2^/day) and found that the insulin-treated group showed a statistically significant mean improvement (IV = 11.84; 95% CI: 0.64–23.04; *p* = 0.04; I2 = 97%) in comparison to the control group. The secondary outcomes showed that the healing time (days) of the wound does not differ statistically (IV = −5.40; 95% CI: −11.28 to 0.48; *p* = 0.07; I2 = 89%); the wound area significantly decreases in the insulin group; no side effects are observed when administering localized insulin; and quality of life significantly improves as the wound heals, regardless of insulin.	[207]
	To pinpoint the causes of wound-healing problems in diabetic patients after transtibial amputation.	Age was a major factor influencing wound-healing issues after TTA in individuals with diabetes, according to statistical analysis (*p* = 0.007). Nevertheless, comorbidities other than diabetes (*p* = 0.209), gender (*p* = 0.677), preoperative anemia (*p* = 0.102), intraoperative blood transfusion (*p* = 0.633), the use of antithrombotic or anticoagulant medications (*p* = 0.556), and the execution of PTA or bypass surgery (*p* = 0.6) did not significantly alter the severity of wound problems. This research concluded that among diabetes patients undergoing TTA, age was a key determinant influencing wound-healing issues.	[208,209,210,211]

## Data Availability

All raw data used in this review are available with the corresponding author on request.
